# P-851. Evaluation of a multifaceted intervention on the management of uncomplicated gram-negative bacteremia

**DOI:** 10.1093/ofid/ofae631.1043

**Published:** 2025-01-29

**Authors:** Katherine C Shihadeh, Michael Deaney, Alex Craig, Margaret M Cooper, Timothy C Jenkins

**Affiliations:** Denver Health, Denver, Colorado; Denver Health Medical Center, Denver, Colorado; Sky Ridge/HealthOne, Denver, Colorado; Denver Health Medical Center, Denver, Colorado; Denver Health, Denver, Colorado

## Abstract

**Background:**

Randomized, controlled trials found 7 days of antibiotics to be as effective as 14 days for the treatment of uncomplicated Gram-negative bacteremia. The routine use of repeat blood cultures for these uncomplicated infections is low yield and not recommended. A review of the management of these infections at Denver Health found the median duration of therapy was 10 days and nearly two-thirds of patients had blood cultures repeated. A multifaceted intervention was implemented to reduce duration of therapy and use of repeat blood cultures. The purpose of this study was to evaluate the impact of the intervention.

**Figure:**
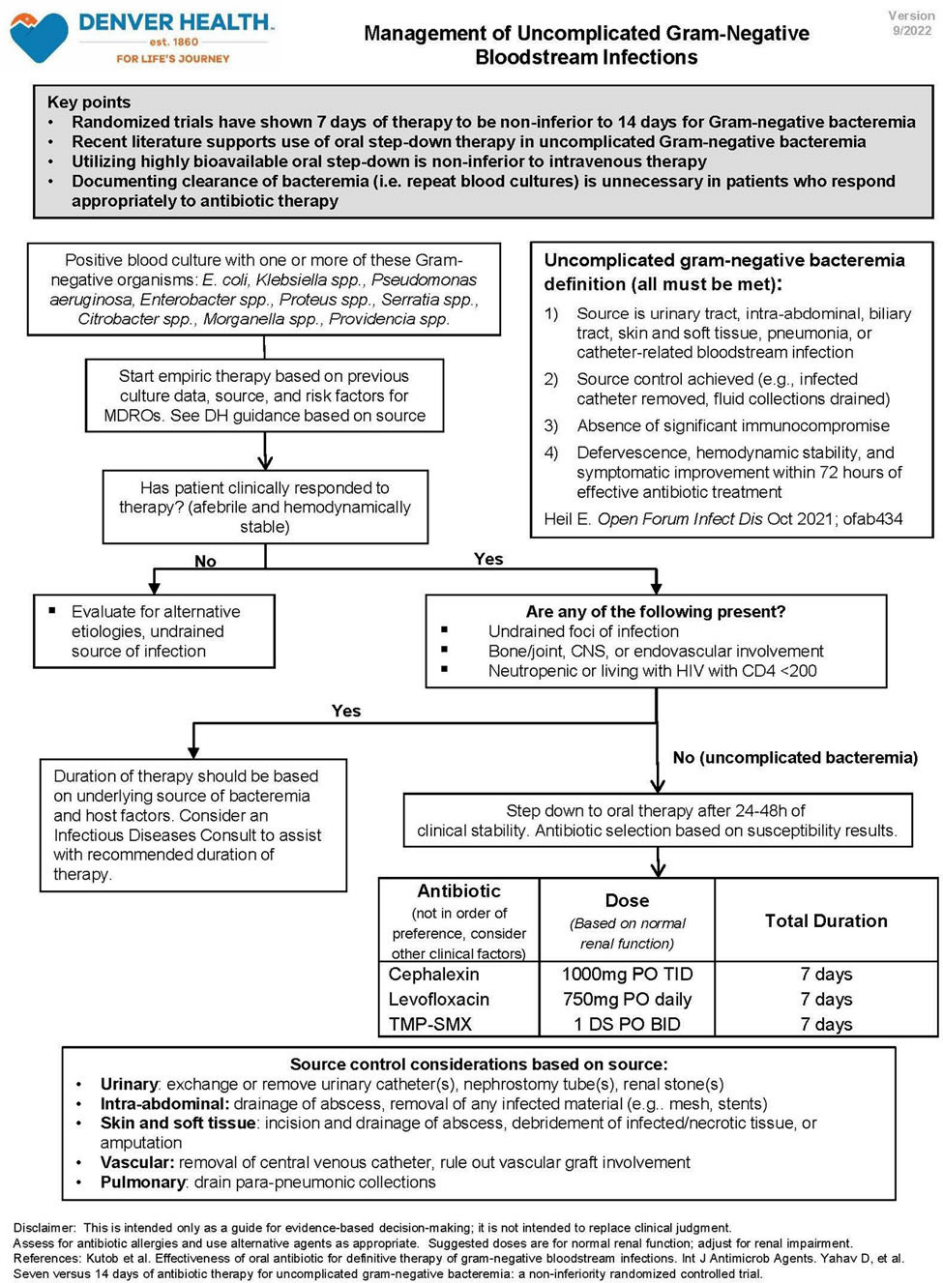

**Methods:**

This retrospective cohort study took place from 1/2019 - 12/2019 (pre-intervention) and 10/2022 - 9/2023 (post-intervention). 3 interventions were implemented including a) development and dissemination of a guideline for the management of uncomplicated Gram-negative bacteremia which recommends 7 days of therapy and against repeat blood cultures for most patients b) clinical decision support to discourage repeat blood cultures c) prospective review of positive blood cultures with provider feedback performed by an infectious diseases pharmacist. All patients over 18 years of age with a positive blood culture with a Gram-negative organism were included. The primary outcome was duration of therapy pre-and post-intervention. The secondary outcome was proportion of patients with repeat blood cultures.

**Figure:**
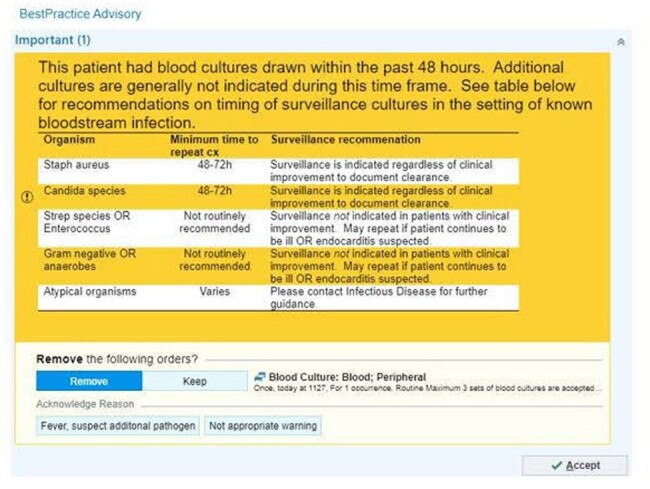

**Results:**

Patient demographics were similar between groups. Urinary tract and *E. coli* were the most common source and pathogen, respectively. Most patients completed therapy with an oral agent; either a fluoroquinolone or cephalosporin. Cefdinir decreased and cephalexin increased based on institutional guidance. The median duration of therapy decreased from 10 to 7 days (p< .01) and fewer patients had repeat blood cultures (60% vs 31%, p< .01). There were fewer cases of *C. difficile* in the post-intervention group, otherwise no differences in safety outcomes.

**Figure:**
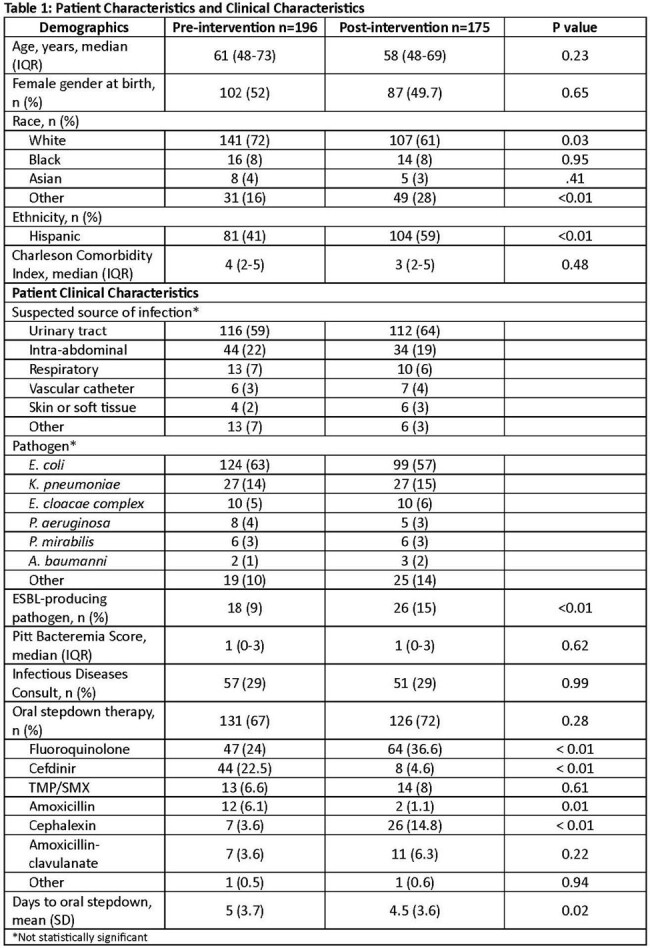

**Conclusion:**

Implementation of a guideline for the management of uncomplicated Gram-negative bacteremia, clinical decision support, and review of positive blood cultures with provider feedback performed by an infectious diseases pharmacist reduced duration of therapy and the use of repeat blood cultures.

**Figure:**
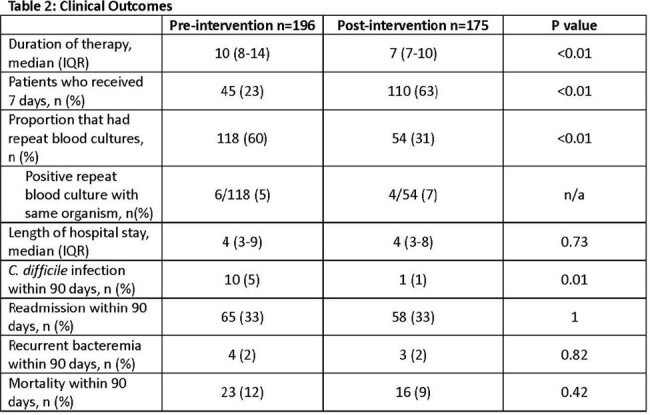

**Disclosures:**

**All Authors**: No reported disclosures

